# From awareness to behaviour: Testing a hierarchy of effects model on the Australian Make Healthy Normal campaign using mediation analysis

**DOI:** 10.1016/j.pmedr.2018.09.003

**Published:** 2018-09-11

**Authors:** James Kite, Joanne Gale, Anne Grunseit, Vincy Li, William Bellew, Adrian Bauman

**Affiliations:** a Prevention Research Collaboration, Sydney School of Public Health and Charles Perkins Centre, The University of Sydney, NSW, Australia; b The Australian Prevention Partnership Centre, Based at Level 6, Charles Perkins Centre, The University of Sydney, NSW, 2006, Australia; c NSW Office of Preventive Health, Liverpool, NSW, Australia

**Keywords:** Mass media campaign, Hierarchy of effects, Overweight and obesity, Mediation

## Abstract

The Make Healthy Normal mass media campaign was a three-year campaign launched in 2015 in New South Wales (NSW), Australia to address community norms around overweight and obesity. It was underpinned by a hierarchy of effects model; a commonly used framework in campaigns but one that has rarely been tested. The campaign evaluation included a cohort study of NSW adults, surveyed three times over 12 months (n = 939 at Wave 3). This study tested the campaign's hierarchy of effects model, which theorized that participants would move from *recognition* to *behaviour change* via *understanding*, *knowledge*, *attitude*, *social norms*, *self*-*efficacy*, and *intention*, using these data. We used the moderation and mediation of effects method proposed by Baron and Kenny, adjusting for age and sex, to test for progression through the hierarchy of effects for two outcomes: physical activity and fast food consumption. We found a clear progression through the theorized model, from *recognition* through to *behaviour change*, via the intermediate variables for both outcomes. We also found several effects not predicted by the theorized model, with consistently strong associations between *understanding* and *attitude*, *understanding* and *self*-*efficacy*, *attitude* and *self*-*efficacy*, and *self*-*efficacy* and *behaviour change* in both outcome models. Our study provides support for the hierarchy of effects as a conceptual model in campaign planning and evaluation of social marketing campaigns. To our knowledge, this is the first study to compare the hierarchy between two behavioural outcomes and the consistency observed between the models adds to the potential usefulness of the hierarchy of effects.

## 1 Introduction

The Make Healthy Normal (MHN)^1^ mass media campaign was launched in June 2015 as part of the strategy to address overweight and obesity in the state of New South Wales (NSW), Australia (Centre for Epidemiology and Evidence, 2016). The campaign ran for three years, using television as the primary media, supported by other channels, including billboards and social media. It was the major communication element of NSW's cross-government approach to obesity prevention, the Healthy Eating and Active Living Strategy (Centre for Population Health, 2013). It challenged the normalisation of being overweight and encouraged adults to adopt healthier lifestyle behaviours, including increasing physical activity and reducing consumption of energy dense, nutrient poor foods. In phase one (2015–2017), the target audience was all NSW adults. Evaluation of phase one found that it was effective at increasing knowledge of physical activity recommendations and the health effects of overweight and obesity but had no effect on behaviour (Kite et al., 2018a).

**Fig. 1:**
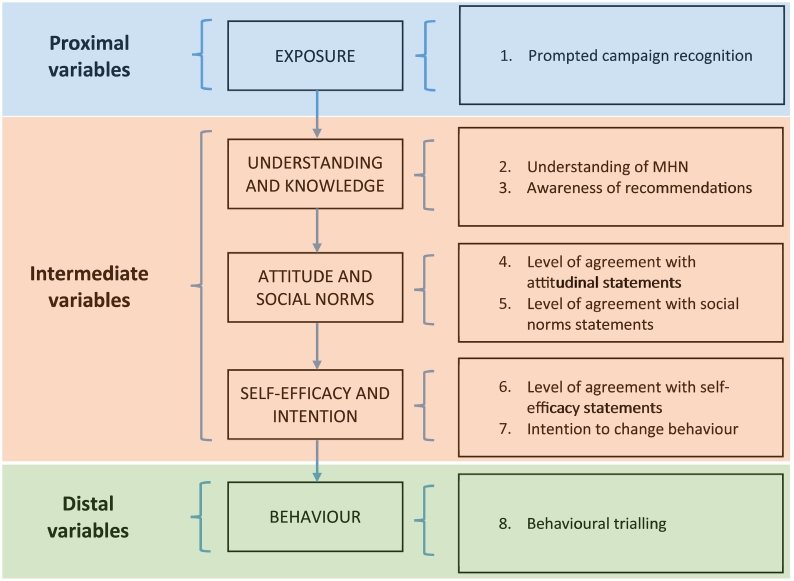
Make Healthy Normal theorized hierarchy of effects model (with evaluation measures, right hand side).

Best practice principles for mass media campaigns suggest that the use of theories or frameworks is important in improving the likelihood of a successful mass media campaign (Grunseit et al., 2016; Noar, 2006; World Health Organization, 2000). Accordingly, MHN's logic model incorporated the hierarchy of effects model (HOEM), shown in Fig. 1, as a central component. However, a recent review of overweight and obesity campaigns found that while many campaigns ostensibly used theories or frameworks in their design and/or evaluation, no campaign reported explicitly testing the underpinning theory or framework (Kite et al., 2018b). Without formal testing, there is no way of knowing whether theories or frameworks are accurate reflections of the constructs they describe, which in turn makes it more difficult to refine and improve the usefulness of public health campaigns.

The HOEM has been recommended for use in public health campaigns since the 1980s (McGuire, 1984), having developed in the 1960s as part of advertising and marketing theory. It posits that proximal variables (e.g. awareness) are causally linked to distal outcomes (e.g. behaviour change) through a series of intermediate measures (e.g. social norms, attitudes, intentions) (Cavill & Bauman, 2004), although the sequence of effects can vary (Barry & Howard, 1990). HOEM also holds that the probability of achieving each outcome decreases as the process moves through the hierarchy, meaning that the proportion of a population that engages in the desired behaviour change would be small.

Within the broader advertising and marketing literature, the HOEM has been tested in the context of sport sponsorship (Alexandris & Tsiotsou, 2012) and digital advertising (Bruner & Kumar, 2000; Yoo et al., 2004; Schlee & Schlee III, 2006), with mixed results. Indeed, Weilbacher (Weilbacher, 2001) has argued that HOEM should be abandoned as an advertising framework because it suffers from several conceptual weaknesses. He believes there has been an uncritical acceptance of HOEM because measurements of HOEM constructs, such as brand awareness, are possible, even though the model itself has not been validated. On the other hand, Barry (Barry, 2002) has argued that the model remains important and that the problem is not, as Weilbacher (Weilbacher, 2001) implies, that the model has not been validated but rather that it is inherently difficult to test. Barry concludes that it is essential to test the model, including investigating different temporal sequences.

Within public health, there is some support for HOEM, although some of the evidence relies on cross-sectional measures (Russell-Bennett et al., 2016). One study using repeat cross-sectional survey data to explore the effects of a radio soap opera on adoption of family planning methods found that the HOEM was useful in predicting the effects of the program in moving people through the stages of change toward adoption (Vaughan & Rogers, 2000). HOEM has also been used as a conceptual framework for understanding the relationship between exposure to junk food marketing and diet and weight without being formally tested (Kelly et al., 2015). Only two studies have examined HOEM using longitudinal data, both investigating physical activity mass media campaigns (Bauman et al., 2008; Craig et al., 2010). Bauman et al. (Bauman et al., 2008) found some support for a hierarchy of effects in the United States-based *VERB* campaign, with awareness and understanding of the campaign's messages (proximal variables) predicting behaviour change (distal outcome) in adolescents, the target audience for *VERB*. However, adolescent attitudes and expectations (both intermediate variables) were not mediators of behaviour change, which would ordinarily be expected based on classical understandings of the HOEM. In their examination of HOEM in an adult population using data from Canada's *ParticipACTION* campaign, Craig et al. (Craig et al., 2010) similarly found support for the model. In this case, however, the results did show that awareness predicted intermediate variables that in turn predicted behaviour change. Collectively, the results of these studies suggest that the HOEM may work differently depending on demographic or other characteristics. Longitudinal studies such as those described above allow investigation of the sequence of HOEM and the limited number of such studies restricts our understanding of how campaigns work.

Hornik (Hornik, 2002) has argued that exposure to messages may affect behaviour by changing social norms – unwritten rules or codes of conduct that govern the way people behave in certain contexts (Chung & Rimal, 2016). Accordingly, social norms are part of several established theories of behaviour change commonly used to inform campaign design and evaluation, including Social Cognitive Theory (Bandura, 1989), Social Learning Theory (Bandura, 1977), and the Theory of Planned Behaviour (Ajzen, 1991). In addition, there have been calls for mass media campaigns to adopt broader social goals, rather than only focusing on the individual (Wakefield et al., 2010; Abroms & Maibach, 2008) and there is clear evidence that social norms have a measurable impact on obesity (Christakis & Fowler, 2007; Shoham et al., 2015). MHN is one of only a handful of campaigns to expressly challenge social norms around diet, physical activity, and weight (Kite et al., 2018b). However, the inclusion of social norms as a step in a campaign's HOEM, has not, to our knowledge, been previously reported.

Explicitly testing a theory or framework is essential if we are to maximise its usefulness through revision or rejection (Rothman, 2004; Nutbeam et al., 2010). To this end, this study aimed to test the Make Healthy Normal HOEM. Specifically, we sought to determine whether (1) the HOEM that underpins the evaluation of MHN is a useful and valid predictive tool; and (2) the cascade of effects in the HOEM varies for different behavioural outcomes. Note that this study does *not* evaluate the campaign's effectiveness, which has been done elsewhere (Kite et al., 2018a).

## 2 Methods

This study drew on data collected for the evaluation of Phase 1 of the MHN campaign, comprising a population-based cohort (i.e. a longitudinal panel) in NSW. The sample was recruited via an online research panel. Participants were invited to complete three online surveys via email in June 2015 (Wave 1 or baseline), March 2016 (Wave 2), and June 2016 (Wave 3). The waves occurred just before the campaign launched, just after the peak television advertising period, and after all television advertising ceased, respectively. To be eligible, participants needed to be 18 years of age or older and living in NSW. Quotas on age, gender, and location were applied at baseline to ensure broad representation of the target audience. In line with the campaign's HOEM (Fig. 1), participants answered questions on their awareness of and response to the campaign, knowledge, attitudes, intentions, and behaviours. In this study, we modelled two obesity-prevention behaviours targeted by the campaign: increasing physical activity and reducing consumption of energy-dense, nutrient-poor snack and fast foods (the latter being defined as food prepared outside of home).

### 2.1 Measures

*Exposure* (or *recognition*) was operationalized as prompted recognition of MHN, assessed by showing each participant the campaign's television commercials and asking if they had seen them before. Participants were then asked if they had seen images from the commercials online, on billboards, bus sides, or at bus stops, in the cinema, or in newspapers or magazines. If a participant answered ‘yes’ to any of the above, they were deemed to have recognized MHN.

*Self*-*assessed understanding* was assessed by asking participants their level of agreement with the statement that MHN was ‘easy to understand’. Participants who agreed, strongly or somewhat, were deemed to have ‘understood’ MHN. This operationalisation of understanding represents a self-assessed judgement of understanding of the campaign.

In both models, the *recognition* and *self*-*assessed understanding* steps in the HOEM were operationalized as described above. The measures used for the remaining steps varied by model and are described in Table 1 and are mapped against the HOEM steps specified in Fig. 1. We included two social norms variables for the physical activity model (*family norms* and *community norms*) but a comparable *family norms* question was not available for the fast food model so only one social norms variable was used in this model. In both models, we opted to use a variable capturing whether participants had tried to change their behaviour as the final outcome, rather than using actual behaviour change. We used this variable because available evidence suggests that one-year of a campaign is unlikely to be enough time for intention to change to behaviour to convert to sustained behaviour change (Kite et al., 2018b), and this way we captured attempted change as well as actual change.

**Table 1:** Measures used to operationalise steps in HOEM models.

HOEM Step	Physical activity model		Fast food model	
	Question	Response options	Question	Response options
Knowledge	To maintain good health, how many minutes of moderate or vigorous physical activity do you think you should do every day? Moderate physical activity can be anything you do that causes a slight increase in your breathing and heart rate for a sustained period such as a brisk walk.	Those who responded 30 min were coded as ‘correct’, in line with Australian Physical Activity Guidelines. All other responses were coded as ‘incorrect’.	Approximately how many kilojoules do you think is the Australian average daily adult intake?	Those who responded between 8000 and 9000 kJ were coded as ‘correct’. All other responses were coded as ‘incorrect’.
Attitude	To what extent do you agree or disagree that making small changes to how physically active you are will decrease your risk of chronic disease	Responses were dichotomised as ‘agree (strongly or somewhat)’ vs. ‘not agree’.	To what extent do you agree or disagree that making small changes to what you eat will decrease your risk of chronic disease	Responses were dichotomised as ‘agree (strongly or somewhat)’ vs. ‘not agree’.
Social Norms	To what extent do you agree or disagree with the following: 1. Most of my family members walk for at least 30 min on almost every day (Family norms) 2. Most people I know walk for at least 30 min on almost every day (Community norms)	Responses were dichotomised as ‘agree (strongly or somewhat)’ vs. ‘not agree’ for both statements.	To what extent do you agree or disagree with the following: More people are avoiding fast food and takeaway snacks to be healthier	Responses were dichotomised as ‘agree (strongly or somewhat)’ vs. ‘not agree’.
Self-efficacy	To what extent do you agree or disagree with the following: I am confident I could increase my physical activity to improve my health	Responses were dichotomised as ‘agree (strongly or somewhat)’ vs. ‘not agree’.	To what extent do you agree or disagree with the following: I am confident I could decrease the amount of fast food or snack food I eat to improve my health	Responses were dichotomised as ‘agree (strongly or somewhat)’ vs. ‘not agree’.
Intention	Do you intend to increase the amount of physical activity you do in the next six months?	Responses were dichotomised as ‘yes, in the next month’ vs. ‘not in the next month’.	To what extent do you think you are likely to decrease or increase your consumption of fast food or snack foods in the next six months?	Responses were dichotomised as ‘likely to decrease’ vs. ‘not likely to decrease’
Behavioural trialling	In the past six months, have you tried to change the amount of moderate or vigorous physical activity that you do?	Responses were dichotomised as ‘tried to increase’ vs. ‘tried to decrease or no change’.	In the last six months, have you tried to decrease the amount of fast food or snack foods that you eat?	Yes vs. no

Note: In all Likert scale responses, ‘neither agree nor disagree’ responses were coded as ‘not agree’. ‘Don't know’ and ‘I'd prefer not to say’ responses were coded as missing.

Likert scale questions were dichotomised (agree vs. not agree) as the distribution of responses was not normal. As we were interested in agreement with the variables, neutral responses were combined with disagree responses. All measures were from Wave 2 of the survey, except for behaviour change, which was from Wave 3.

### 2.2 Statistical analysis

We conducted a series of logistic regression models to test progression through the theorized HOEM, using the moderation and mediation of effects method proposed by Baron and Kenny (Baron & Kenny, 1986) and used by Craig et al. (Craig et al., 2010) in their test of ParticipACTION's HOEM. Specifically, we tested for mediation by introducing each of the steps in the theorized HOEM sequentially, controlling for each of the intervening variables. For example, the association between *recognition* and *attitude* was tested while controlling for *self*-*assessed understanding* and *knowledge*. We used Holm adjustments applied during inference to minimise Type 1 errors due to the number of tests required (Bender & Lange, 2001; Aickin & Gensler, 1996). A variable was determined to be a mediator if three conditions were met: (1) it was significantly associated with the independent variable; (2) it predicted the outcome variable; and (3) when both the independent and proposed mediator were included in the model, any association between the independent variable and the outcome variable became non-significant (full mediation) or the odds ratio moved closer to non-significance (partial mediation). We modified our approach from that of Baron and Kenny by testing for mediation regardless of whether there was a significant association between the independent and outcome variables in models without the proposed mediator, as this has been highlighted as a significant limitation of this method (Hayes, 2009). While we acknowledge the relative strengths and weaknesses of other methods compared to Baron and Kenny's method, we note that the different methods have been shown to agree >90% of the time (Hayes & Scharkow, 2013).

As we included two social norms variables in the physical activity model and did not hypothesise that one would precede the other, all results for *family norms* and *community norms* have been adjusted for the other variable. We included age and sex as covariates in all models and set an alpha threshold of 0.05 for statistical significance. Only significant relationships are included in the final models. Participants with missing data were excluded from the analysis.

We also conducted sensitivity analyses to test whether baseline behaviour influenced the models. In these analyses, we excluded participants who were meeting Australian physical activity guidelines at baseline (Department of Health, 2014) and those who ate fast food or snack foods less than once a day at baseline, respectively. The latter grouping was chosen to reflect the Australian dietary guidelines, which recommend that the consumption of such foods be limited (National Health and Medical Research Council (NHMRC), 2013).

All analyses were conducted in SAS Version 9.4.

## 3 Results

In total, 2259 participants completed the baseline survey, with 1225 completing Wave 2 and 1113 Wave 3. Just over half the sample (approximately 53%) at each time point were female, and two-thirds (66%) were aged over 40 years at baseline, increasing to 76% at Waves 2 and 3. Almost two-fifths (39%) of the sample failed to meet Australian physical activity recommendations and over one-third (35%) consumed one or more serves of fast food or snack foods per day at baseline. The complete demographics of the sample are available elsewhere (Kite et al., 2018a). The prevalence of the modelled variables is shown in Table 2.

**Table 2:** Prevalence of modelled variables at Wave 2 or Wave 3 (behaviour only).

HOEM Step	Variable	N (%)
Exposure	Prompted recognition of MHN	
	Yes	432 (35)
	No	772 (63)
	Missing	21 (2)
Understanding and knowledge	MHN is ‘easy to understand’	
	Agree	863 (70)
	Not agree	329 (27)
	Missing	33 (3)
	Knowledge of physical activity guidelines	
	Correct	578 (47)
	Incorrect	645 (53)
	Missing	2 (0)
	Knowledge of average daily kilojoule intake for adults	
	Correct	172 (15)
	Incorrect	933 (83)
	Missing	14 (1)
Attitude and social norms	Making small changes to how physically active you are will decrease your risk of chronic disease	
	Agree	996 (81)
	Not agree	219 (18)
	Missing	10 (1)
	Making small changes to what you eat will decrease your risk of chronic disease	
	Agree	977 (80)
	Not agree	238 (19)
	Missing	10 (1)
	Most of my family members walk for at least 30 min on almost every day	
	Agree	439 (36)
	Not agree	764 (62)
	Missing	22 (2)
	Most people I know walk for at least 30 min on almost every day	
	Agree	280 (23)
	Not agree	924 (75)
	Missing	21 (2)
	More people are avoiding fast food and takeaway snacks to be healthier	
	Agree	460 (38)
	Not agree	751 (61)
	Missing	14 (1)
Self-efficacy and intention	I am confident I could increase my physical activity to improve my health	
	Agree	782 (64)
	Not agree	432 (35)
	Missing	11 (1)
	I am confident I could decrease the amount of fast food or snack food I eat to improve my health	
	Agree	700 (57)
	Not agree	503 (41)
	Missing	22 (2)
	Intend to increase the amount of physical activity I do in the next month	
	Yes	343 (31)
	Not in the next month	763 (69)
	Missing	7 (1)
	Likely to decrease consumption of fast food or snack foods in the next six months	
	Yes	437 (39)
	No	654 (59)
	Missing	24 (2)
Behavioural trialling	Tried to increase the amount of moderate or vigorous physical activity	
	Yes	578 (52)
	No	530 (48)
	Missing	5 (0)
	Tried to decrease the amount of fast food or snack foods consumed	
	Yes	471 (42)
	No	623 (56)
	Missing	19 (2)

**Fig. 2:**
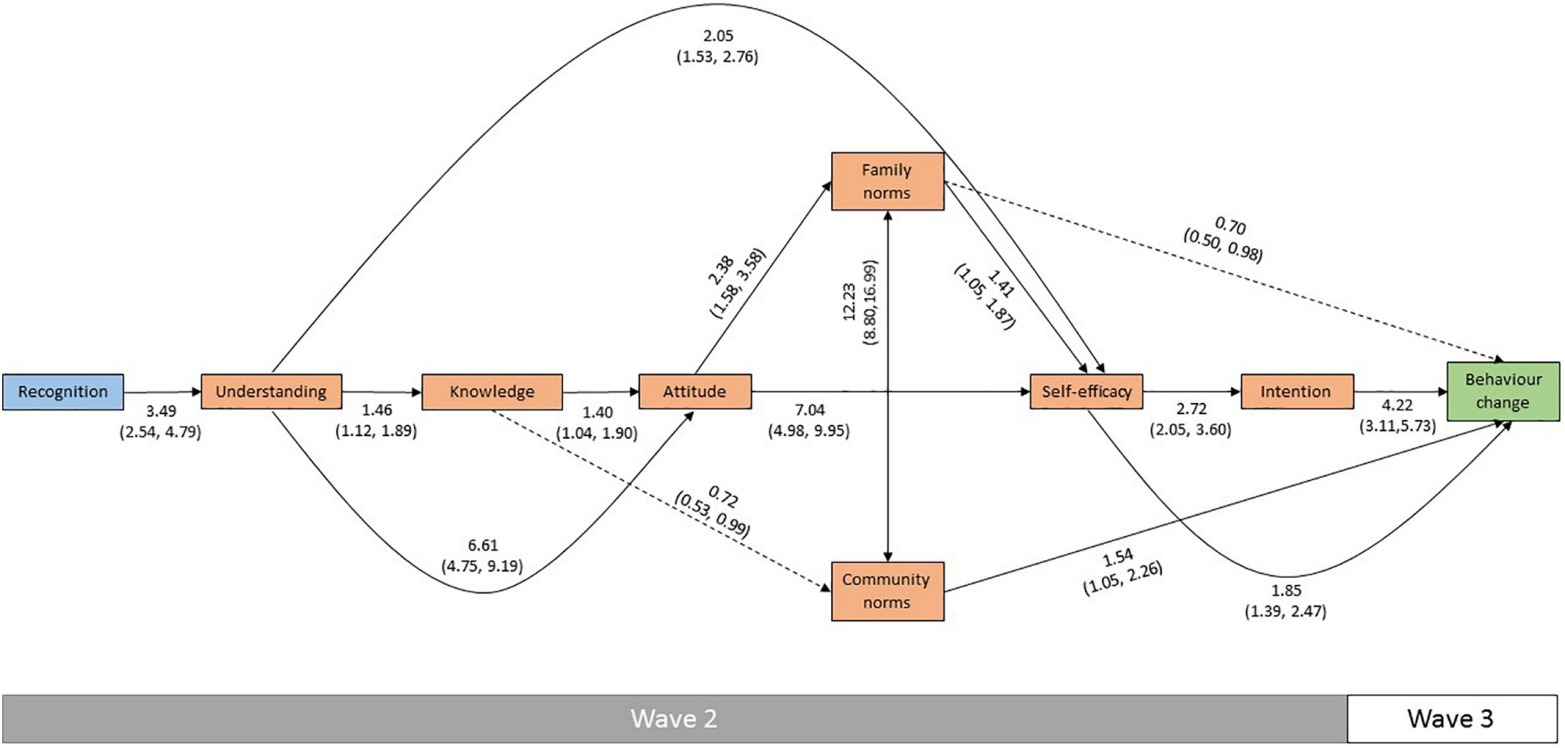
Hierarchy of effects model predicting an attempted increase in physical activity, showing adjusted odds ratios with 95% confidence intervals. Note: Figure shows statistically significant associations (p < 0.05) only. Model adjusted for age and sex.

**Fig. 3:**
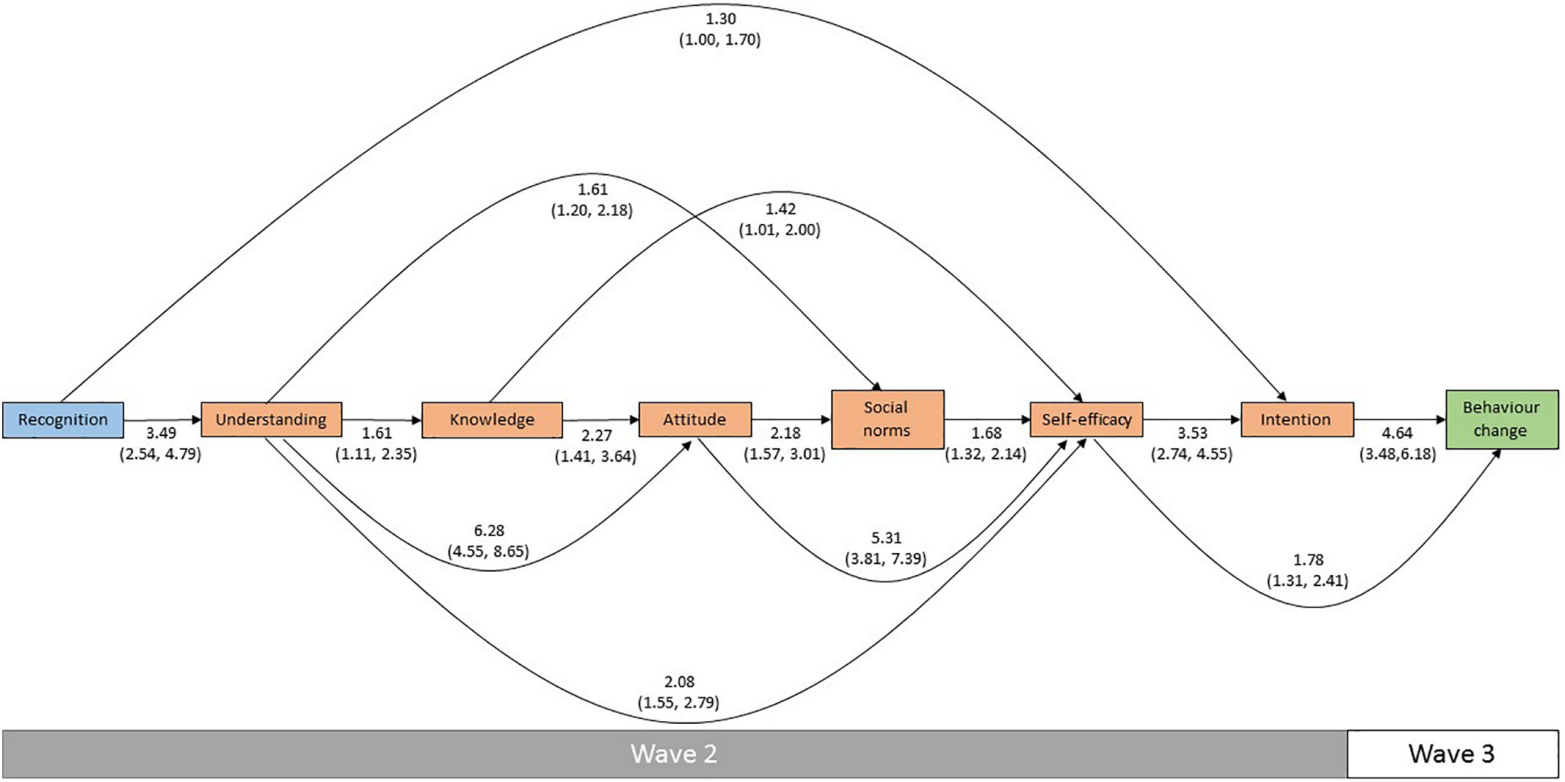
Hierarchy of effects model predicting an attempted reduction in fast food consumption, showing adjusted odds ratios with 95% confidence intervals. Note: Figure shows statistically significant associations (p < 0.05) only. Model adjusted for age and sex.

A clear path from *recognition* to *behaviour change* through the intermediate variables was evident in both the model predicting an attempted increase in physical activity (Fig. 2 and Supplementary Table 1) and the model predicting a reduction in consumption of fast food (Fig. 3 and Supplementary Table 2). Each step in both models had a significant positive association with the step immediately preceding it, in line with the theorized HOEM, with two exceptions: *attitude* did not predict *community norms* and *community norms* did not predict *self*-*efficacy* in the physical activity model. These associations were instead fully mediated by the associations between *attitude* and *family norms* and *family norms* and *self*-*efficacy*. After applying Holm adjustments, the associations between *understanding* and *knowledge* became non-significant in both models, while in the physical activity model, the associations between *knowledge* and *attitude* and between *family norms* and *self*-*efficacy* also became non-significant.

Several significant effects that were not predicted by the theorized HOEM (that is, they were not fully mediated by the interim variables) were evident in the models. We observed positive associations between *understanding* and *attitude*, *understanding* and *self*-*efficacy*, *attitude* and *self*-*efficacy*, and *self*-*efficacy* and *behaviour change* in both models. In the case of *understanding* and *attitude* and *attitude* and *self*-*efficacy*, the interim variables did not appear to have any mediating effect on their associations in either model. We also observed some differences between the models. In the physical activity model, we found a significant positive association between *community norms* and *behaviour change* and significant inverse associations between *knowledge* and *community norms* and *family norms* and *behaviour change*, although these became non-significant after applying Holm adjustments. In the fast food model, we found a significant positive association between *understanding* and *social norms*. We also found associations between *recognition* and *intention*, and *knowledge* and *self*-*efficacy*, although these became non-significant after applying Holm adjustments.

Sensitivity analyses showed broadly consistent results compared to the models including all participants (Supplementary Tables 3 and 4). The one notable exception was that in the physical activity model all associations between both *family norms* and *community norms* and the other variables became non-significant, although the directions of effect remained consistent with the full models.

## 4 Discussion

Our results provide some support for the HOEM as a theoretical framework for explaining mass media campaigns. Additionally, we have shown that the cascade of effects in the HOEM is broadly consistent across two separate outcomes: physical activity and consumption of snack or fast foods. This suggests that the mechanisms described in the HOEM are useful in describing campaign effects, as well as supports the use of HOEM in campaign planning and evaluation. This adds more to the theoretical evidence base underpinning campaigns, and is in line with best practice principles which recommend an increase in the use of theory and frameworks in mass media campaigns (Grunseit et al., 2016; World Health Organization, 2000; Randolph & Viswanath, 2004).

Notably, there were very strong associations between *understanding* and *attitude* and *attitude* and *self*-*efficacy* in both models. While this study did not set out to evaluate MHN and we cannot conclude that these associations are causal, this, coupled with the results from evaluation of Phase 1 (Kite et al., 2018a), suggests that MHN may effectively convey the need to make small changes to lifestyle to benefit health and that accepting such messages gives people confidence that they can make changes. Further, the fact that these associations were not mediated by the interim variables suggests that campaign messages relating to *knowledge* and *social norms* could be afforded lesser attention in similar campaigns, particularly when limited resources are available. This is supported by an earlier review we did of overweight and obesity prevention mass media campaigns, which found that campaigns with formative evaluation consistently found that knowledge of the health effects and of behaviours was relatively high before implementation (Kite et al., 2018b). We therefore argue that, following rigorous formative evaluation, future campaigns could focus less on such messages and more on behaviour change.

As mentioned above, there have been calls for mass media campaigns to move beyond focusing exclusively on the individual and adopt broader social goals (Wakefield et al., 2010; Abroms & Maibach, 2008), although to date few obesity prevention campaigns have done so (Kite et al., 2018b). Our inclusion of social norms is therefore innovative in the obesity prevention context, considering that most previous social norms campaign research relates to alcohol reduction campaigns in college students in the United States (Wechsler et al., 2003; DeJong et al., 2006; Foxcroft et al., 2015). While there is evidence of correlations between social norms and obesity (Shoham et al., 2015; Burke et al., 2010; Lynch et al., 2009), our analysis is one of the first to show longitudinal associations between social norms and trialling of behaviour change.

In the physical activity model, we were able to divide social norms into two variables: *family norms* and *community norms*. Although we did not hypothesise that one of these variables would precede the other, the lack of associations between *community norms* and *attitude* and *self*-*efficacy* (the immediately preceding and following steps in the HOEM) suggests that changing *family norms* is a first step before changing *community norms*. This may reflect the social and environmental context within which an individual's behaviour takes place, as described by the social ecological model (McLeroy et al., 1988). That is, a participant might be aware of the benefits of increasing how physically active and feel that their immediate family is supportive of physical activity, but also of environmental barriers, such as working hours and urban design, which may inhibit their personal ability to enact behaviour change. It also appears that *family norms* may be more important for progression in the HOEM as *community norms* was not associated with *self*-*efficacy*. Alternatively, this pattern of associations may be due to *community norms* being on a different cognitive path to *attitude* and *self*-*efficacy*, which are cognitively individualised attributes, while *community norms* is an externalised cognition shared across a community. Further research on the role of social norms in these campaigns is warranted, especially considering that many of the associations became non-significant after Holm adjustment and in our sensitivity analysis.

Our findings support those of the ParticipACTION campaign in that the intermediate variables did play a role in the hierarchy (Craig et al., 2010), in contrast to VERB (Bauman et al., 2008). Given VERB's findings, further analyses in children and adolescents are necessary to explore the cognitive processes and relevance of HOEM for adolescents, compared to adults. It will also be important to explore whether there are differences by other demographic or behavioural characteristics, given that for ParticipACTION, inactive and active participants had varying cascades of effects through the hierarchy, with the model a better fit for inactive participants. Our modelling, conversely, showed broadly consistent effects for both physical activity and snack and fast food consumption, regardless of baseline behaviour. That the models were broadly comparable for both behavioural outcomes may indicate that the concept of a hierarchy of effects is robust across behaviours but further analyses for other behaviours will be necessary to confirm this.

A limitation of this study is that we are demonstrating associations, rather than causal pathways. Nonetheless, as discussed above, our findings are suggestive of mechanisms that could be useful in campaign planning and implementation. While our use of longitudinal data was a strength, ideally, the study would have had more than two waves of follow-up as that would have given us more scope to explore the associations over time. However, that we were able to go beyond physical activity and examine the HOEM for fast food consumption is a strength of this study. Next, the mediation analysis requires a high number of tests, increasing the likelihood of Type 1 error (Pollard & Richardson, 1987). However, the application of Holm adjustments did not significantly alter the nature of the final models. Further, we cannot be sure that the variables used in this study are measuring the latent concepts to which we assigned them. Future studies should consider testing their measures as part of formative evaluation to ensure that the measures capture what is intended. The high loss to follow-up between Wave 1 and 2 may also have introduced bias, although sensitivity analyses conducted for an earlier study (Kite et al., 2018a) suggest our findings are robust. Finally, we were not able to model factors outside the immediate campaign, such as supportive community programs and infrastructure, such as the broader implementation of the Healthy Eating and Active Living Strategy, that should form part of all comprehensive mass media campaigns (Hastings, 2007; Hoek & Jones, 2011). Therefore our results could not assess the influence of these important external factors.

## 5 Conclusions

To our knowledge, this is the first study to test a HOEM for two separate health behavioural outcomes. Our finding that the cascade of effects were broadly consistent between the two outcomes adds further weight to the usefulness of HOEM as a campaign planning and evaluation framework. Further testing of HOEM is needed in campaigns targeting a wider variety of behaviours and outcomes. Nonetheless, this study has provided support for the use of HOEM in mass media campaign planning.

## Appendix A Supplementary data

Supplementary tables showing direct and fully adjusted effects for full models and sensitivity analyses. Image 1

## Footnotes

1 Abbreviations: HOEM – Hierarchy of effects model; MHN – Make Healthy Normal campaign.
