# Supplementary material for: From awareness to behaviour: Testing a hierarchy of effects model on the Australian Make Healthy Normal campaign using mediation analysis
Source: Prev Med Rep. 2018 Sep 11;12:140–7. doi: 10.1016/j.pmedr.2018.09.003 (PMC6152809; doi:10.1016/j.pmedr.2018.09.003)
Supplement: Supplementary file 1 — Supplementary tables showing direct and fully adjusted effects for full models and sensitivity analyses. [file mmc1.docx]

Supplementary Table 1 Direct and fully adjusted effects for all outcome variables in the physical activity model

| **Independent variable** | **Outcome variable** | **Direct effect** | | **Effect after adjusting for all interim variables** | |
| --- | --- | --- | --- | --- | --- |
|  |  | **Odds ratio (95% CI)** | **P value** | **Odds ratio (95% CI)** | **P value** |
| **Recognition** | Understanding | 3.49 (2.54, 4.79) | <0.001 |  |  |
|  | Knowledge | 1.22 (0.96, 1.55) | 0.103 | 1.13 (0.88, 1.44) | 0.346 |
|  | Attitude | 1.49 (1.07, 2.08) | 0.018 | 0.90 (0.62, 1.31) | 0.589 |
|  | Family norms | 1.01 (0.79, 1.29) | 0.947 | 0.89 (0.67, 1.20) | 0.456 |
|  | Community norms | 1.07 (0.81, 1.42) | 0.628 | 1.05 (0.75, 1.46) | 0.785 |
|  | Self-efficacy | 1.45 (1.13, 1.87) | 0.004 ^a^ | 1.16 (0.87, 1.54) | 0.310 |
|  | Intention | 1.27 (0.98, 1.63) | 0.067 | 1.18 (0.90, 1.55) | 0.224 |
|  | Behaviour change | 1.39 (1.06, 1.83) | 0.018 ^a^ | 1.22 (0.89, 1.65) | 0.215 |
| **Understanding** | Knowledge | 1.46 (1.12, 1.89) | 0.004^a^ |  |  |
|  | Attitude | 6.74 (4.85, 9.36) | <0.001 | 6.61 (4.75, 9.19) | <0.001 |
|  | Family norms | 1.61 (1.22, 2.12) | 0.001 | 1.21 (0.86, 1.69) | 0.268 |
|  | Community norms | 1.50 (1.08, 2.07) | 0.015 ^a^ | 1.14 (0.78, 1.68) | 0.493 |
|  | Self-efficacy | 3.25 (2.49, 4.24 | <0.001 | 2.05 (1.53, 2.76) | <0.001 |
|  | Intention | 1.33 (1.00, 1.77) | 0.047 ^a^ | 1.00 (0.73, 1.37) | 0.984 |
|  | Behaviour change | 1.25 (0.94, 1.68) | 0.130 | 0.89 (0.63, 1.25) | 0.498 |
| **Knowledge** | Attitude | 1.40 (1.04, 1.90) | 0.029 ^a^ |  |  |
|  | Family norms | 0.78 (0.61, 0.98) | 0.036 ^a^ | 0.85 (0.65, 1.12) | 0.241 |
|  | Community norms | 0.69 (0.53, 0.91) | 0.009 ^a^ | 0.72 (0.53, 0.99) | 0.044 ^a^ |
|  | Self-efficacy | 1.32 (1.04, 1.68) | 0.021 ^a^ | 1.25 (0.96, 1.62) | 0.095 |
|  | Intention | 1.08 (0.84, 1.38) | 0.545 | 1.08 (0.84, 1.39) | 0.558 |
|  | Behaviour change | 1.33 (1.02, 1.72) | 0.032 ^a^ | 1.27 (0.95, 1.68) | 0.103 |
| **Attitude** | Family norms | 2.88 (2.00, 4.17) | <0.001 | 2.38 (1.58, 3.58) | <0.001 |
|  | Community norms | 2.47 (1.61, 3.80) | <0.001 | 1.60 (0.98, 2.60) | 0.059 |
|  | Self-efficacy | 7.77 (5.54, 10.91) | <0.001 | 7.04 (4.98, 9.95) | <0.001 |
|  | Intention | 1.53 (1.10, 2.15) | 0.013 ^a^ | 0.89 (0.61, 1.31) | 0.564 |
|  | Behaviour change | 1.74 (1.24 ,2.45) | 0.001 | 1.18 (0.79, 1.76) | 0.416 |
| **Family norms** ^b^ | Community norms | 12.23 (8.80, 16.99) | <0.001 |  |  |
|  | Self-efficacy | 1.57 (1.22, 2.02) | 0.001 | 1.41 (1.05, 1.87) | 0.020 ^a^ |
|  | Intention | 1.46 (1.14, 1.88) | 0.003 | 1.20 (0.89, 1.61) | 0.233 |
|  | Behaviour change | 1.03 (0.78, 1.34) | 0.845 | 0.70 (0.50, 0.98) | 0.036 ^a^ |
| **Community norms** ^b^ | Family norms | 12.22 (8.80, 16.99) | <0.001 |  |  |
|  | Self-efficacy | 1.53 (1.14, 2.06) | 0.005 ^a^ | 1.28 (0.91, 1.79) | 0.152 |
|  | Intention | 1.55 (1.17, 2.06) | 0.002 | 1.32 (0.95, 1.83) | 0.103 |
|  | Behaviour change | 1.42 (1.04, 1.95) | 0.027 ^a^ | 1.54 (1.05, 2.26) | 0.029 ^a^ |
| **Self-efficacy** | Intention | 2.72 (2.05, 3.60) | <0.001 |  |  |
|  | Behaviour change | 2.30 (1.75, 3.03) | <0.001 | 1.85 (1.39, 2.47) | <0.001 |
| **Intention** | Behaviour change | 4.22 (3.11 ,5.73) | <0.001 |  |  |

^a^ Becomes non-significant after Holm adjustment.

^b^ All results for family norms and community norms are adjusted for the other norms variable.

Supplementary Table 2 Direct and fully adjusted effects for all outcome variables in the fast food model

| **Independent variable** | **Outcome variable** | **Direct effect** | | **Effect adjusting for all interim variables** | |
| --- | --- | --- | --- | --- | --- |
|  |  | **Odds ratio (95% CI)** | **P value** | **Odds ratio (95% CI)** | **P value** |
| **Recognition** | Understanding | 3.49 (2.54, 4.79) | <0.001 |  |  |
|  | Knowledge | 1.42 (1.04, 1.93) | 0.028 ^a^ | 1.34 (0.97, 1.84) | 0.075 |
|  | Attitude | 2.14 (1.53, 3.00) | <0.001 | 1.43 (0.98, 2.07) | 0.061 |
|  | Social norms | 1.38 (1.09, 1.76) | 0.009 ^a^ | 1.16 (0.90, 1.50) | 0.243 |
|  | Self-efficacy | 1.70 (1.32, 2.17) | <0.001 | 1.25 (0.95, 1.64) | 0.106 |
|  | Intention | 1.51 (1.19, 1.92) | 0.001 | 1.30 (1.00, 1.70) | 0.049 ^a^ |
|  | Behaviour change | 1.41 (1.06, 1.86) | 0.016 ^a^ | 1.20 (0.87, 1.65) | 0.258 |
| **Understanding** | Knowledge | 1.61 (1.11, 2.35) | 0.013 ^a^ |  |  |
|  | Attitude | 6.12 (4.47, 8.40) | <0.001 | 6.28 (4.55, 8.65) | <0.001 |
|  | Social norms | 1.97 (1.49, 2.61) | <0.001 | 1.61 (1.20, 2.18) | 0.002 |
|  | Self-efficacy | 3.14 (2.40, 4.12) | <0.001 | 2.08 (1.55, 2.79) | <0.001 |
|  | Intention | 1.42 (1.09, 1.86) | 0.010 ^a^ | 1.03 (0.76, 1.39) | 0.863 |
|  | Behaviour change | 1.28 (0.94, 1.72) | 0.113 | 0.92 (0.64, 1.31) | 0.628 |
| **Knowledge** | Attitude | 2.27 (1.41, 3.64) | 0.001 |  |  |
|  | Social norms | 1.45 (1.06, 1.97) | 0.020 ^a^ | 1.33 (0.97, 1.82) | 0.073 |
|  | Self-efficacy | 1.69 (1.22, 2.34) | 0.002 | 1.42 (1.01, 2.00) | 0.045 ^a^ |
|  | Intention | 0.99 (0.73, 1.35) | 0.947 | 0.82 (0.59, 1.14) | 0.232 |
|  | Behaviour change | 1.19 (0.82, 1.72) | 0.363 | 1.14 (0.76, 1.71) | 0.533 |
| **Attitude** | Social norms | 2.18 (1.57, 3.01) | <0.001 |  |  |
|  | Self-efficacy | 5.65 (4.06, 7.85) | <0.001 | 5.31 (3.81, 7.39) | <0.001 |
|  | Intention | 1.83 (1.34, 2.50) | <0.001 | 1.08 (0.77, 1.53) | 0.644 |
|  | Behaviour change | 1.57 (1.11, 2.23) | 0.011 ^a^ | 1.09 (0.73, 1.63) | 0.671 |
| **Social norms** | Self-efficacy | 1.68 (1.32, 2.14) | <0.001 |  |  |
|  | Intention | 1.47 (1.16, 1.86) | 0.002 | 1.27 (0.99, 1.64) | 0.057 |
|  | Behaviour change | 1.38 (1.05, 1.81) | 0.021 ^a^ | 1.20 (0.89, 1.61) | 0.237 |
| **Self-efficacy** | Intention | 3.53 (2.74, 4.55) | <0.001 |  |  |
|  | Behaviour change | 2.51 (1.90, 3.33) | <0.001 | 1.78 (1.31, 2.41) | <0.001 |
| **Intention** | Behaviour change | 4.64 (3.48, 6.18) | <0.001 |  |  |

^a^ Becomes non-significant after Holm adjustment.

Supplementary Table 3 Sensitivity analysis for physical activity model, including only participants not meeting Australian physical activity guidelines at baseline

| **Independent variable** | **Outcome variable** | **Direct effect** | | **Effect after adjusting for all interim variables** | |
| --- | --- | --- | --- | --- | --- |
|  |  | **Odds ratio (95% CI)** | **P value** | **Odds ratio (95% CI)** | **P value** |
| **Recognition** | Understanding | 5.51 (3.20, 9.49) | <0.001 |  |  |
|  | Knowledge | 1.39 (0.95, 2.02) | 0.086 | 1.10 (0.74, 1.64) | 0.638 |
|  | Attitude | 2.43 (1.43, 4.13) | 0.001 | 1.28 (0.71, 2.31) | 0.416 |
|  | Family norms | 1.37 (0.90, 2.08) | 0.139 | 1.25 (0.80, 1.95) | 0.323 |
|  | Community norms | 1.30 (0.81, 2.08) | 0.275 | 1.05 (0.55, 2.01) | 0.875 |
|  | Self-efficacy | 1.56 (1.06, 2.27) | 0.022 ^a^ | 0.88 (0.56, 1.38) | 0.577 |
|  | Intention | 0.92 (0.60, 1.40) | 0.688 | 0.70 (0.43, 1.12) | 0.132 |
|  | Behaviour change | 1.27 (0.83, 1.95) | 0.265 | 1.17 (0.72, 1.92) | 0.526 |
| **Understanding** | Knowledge | 2.22 (1.45, 3.39) | <0.001 |  |  |
|  | Attitude | 6.95 (4.19, 11.52) | <0.001 | 6.48 (3.88, 10.81) | <0.001 |
|  | Family norms | 1.34 (0.83, 2.17) | 0.230 | 1.11 (0.67, 1.87) | 0.680 |
|  | Community norms | 1.23 (0.72, 2.12) | 0.450 | 1.09 (0.53, 2.25) | 0.820 |
|  | Self-efficacy | 4.59 (2.97, 7.09) | <0.001 | 3.07 (1.91, 4.94) | <0.001 |
|  | Intention | 1.77 (1.09, 2.88) | 0.022 ^a^ | 1.03 (0.59, 1.80) | 0.920 |
|  | Behaviour change | 1.60 (1.01, 2.53) | 0.047 ^a^ | 0.97 (0.56, 1.67) | 0.913 |
| **Knowledge** | Attitude | 1.98 (1.24, 3.18) | 0.005 ^a^ |  |  |
|  | Family norms | 1.07 (0.71, 1.62) | 0.738 | 1.00 (0.66, 1.53) | 0.982 |
|  | Community norms | 0.82 (0.52, 1.32) | 0.418 | 0.64 (0.35, 1.17) | 0.144 |
|  | Self-efficacy | 1.51 (1.05, 2.18) | 0.026 ^a^ | 1.25 (0.84, 1.86) | 0.280 |
|  | Intention | 1.24 (0.83, 1.86) | 0.297 | 1.15 (0.74, 1.78) | 0.530 |
|  | Behaviour change | 1.24 (0.82, 1.86) | 0.309 | 1.18 (0.75, 1.83) | 0.474 |
| **Attitude** | Family norms | 2.18 (1.18, 4.04) | 0.013 ^a^ | 1.78 (0.84, 3.77) | 0.131 |
|  | Community norms | 1.93 (0.97, 3.84) | 0.059 | 1.50 (0.61, 3.65) | 0.376 |
|  | Self-efficacy | 9.17 (5.22, 16.12) | <0.001 | 8.52 (4.80, 15.13) | <0.001 |
|  | Intention | 2.88 (1.55, 5.34) | 0.001 | 1.58 (0.79, 3.18) | 0.197 |
|  | Behaviour change | 2.40 (1.41, 4.08) | 0.001 | 1.54 (0.84, 2.84) | 0.164 |
| **Family norms** ^b^ | Community norms | 34.11 (17.74, 65.58) | <0.001 |  |  |
|  | Self-efficacy | 1.14 (0.75, 1.72) | 0.551 | 0.90 (0.53, 1.52) | 0.689 |
|  | Intention | 1.85 (1.19, 2.89) | 0.006 ^a^ | 1.32 (0.72, 2.42) | 0.375 |
|  | Behaviour change | 1.43 (0.90, 2.27) | 0.127 | 0.90 (0.48, 1.66) | 0.734 |
| **Community norms** ^b^ | Family norms | 34.07 (17.73, 65.47) | <0.001 |  |  |
|  | Self-efficacy | 1.39 (0.86, 2.24) | 0.181 | 1.53 (0.83, 2.81) | 0.171 |
|  | Intention | 2.29 (1.41, 3.72) | 0.001 | 1.83 (0.94, 3.55) | 0.075 |
|  | Behaviour change | 1.77 (1.04, 3.00) | 0.034 ^a^ | 1.56 (0.77, 3.17) | 0.216 |
| **Self-efficacy** | Intention | 3.79 (2.38, 6.04) | <0.001 |  |  |
|  | Behaviour change | 2.24 (1.47, 3.40) | <0.001 | 1.72 (1.10, 2.68) | 0.017 ^a^ |
| **Intention** | Behaviour change | 3.84 (2.38, 6.21) | <0.001 |  |  |

^a^ Becomes non-significant after Holm adjustment.

^b^ All results for family norms and community norms are adjusted for the other norms variable.

Supplementary Table 4 Sensitivity analysis for fast food model, including only participants consuming 1 or more serves of fast food or snack food per day at baseline

| **Independent variable** | **Outcome variable** | **Direct effect** | | **Effect adjusting for all interim variables** | |
| --- | --- | --- | --- | --- | --- |
|  |  | **Odds ratio (95% CI)** | **P value** | **Odds ratio (95% CI)** | **P value** |
| **Recognition** | Understanding | 2.32 (1.39, 3.89) | 0.001 |  |  |
|  | Knowledge | 2.14 (1.25, 3.68) | 0.006 ^a^ | 2.13 (1.22, 3.71) | 0.008 ^a^ |
|  | Attitude | 2.74 (1.57, 4.77) | <0.001 | 2.13 (1.16, 3.91) | 0.015 |
|  | Social norms | 1.42 (0.92, 2.17) | 0.111 | 1.23 (0.79, 1.93) | 0.360 |
|  | Self-efficacy | 1.55 (1.01, 2.37) | 0.046 ^a^ | 1.01 (0.62, 1.64) | 0.982 |
|  | Intention | 1.05 (0.69, 1.58) | 0.823 | 0.87 (0.55, 1.36) | 0.534 |
|  | Behaviour change | 1.22 (0.77, 1.94) | 0.400 | 1.17 (0.71, 1.94) | 0.534 |
| **Understanding** | Knowledge | 1.68 (0.85, 3.32) | 0.135 |  |  |
|  | Attitude | 7.21 (4.16, 12.5) | <0.001 | 7.24 (4.14,12.65) | <0.001 |
|  | Social norms | 2.61 (1.51, 4.52) | 0.001 | 2.25 (1.24,4.05) | 0.007 ^a^ |
|  | Self-efficacy | 3.99 (2.43, 6.56) | <0.001 | 2.14 (1.22,3.77) | 0.008 ^a^ |
|  | Intention | 1.73 (1.07, 2.81) | 0.027 ^a^ | 1.15 (0.66,1.99) | 0.620 |
|  | Behaviour change | 1.18 (0.69, 2.01) | 0.537 | 0.88 (0.48,1.62) | 0.683 |
| **Knowledge** | Attitude | 2.78 (1.25, 6.19) | 0.012 ^a^ |  |  |
|  | Social norms | 1.11 (0.64, 1.92) | 0.713 | 1.00 (0.57, 1.75) | 0.995 |
|  | Self-efficacy | 2.65 (1.40, 4.99) | 0.003 ^a^ | 2.17 (1.10, 4.27) | 0.025 ^a^ |
|  | Intention | 1.12 (0.66, 1.91) | 0.676 | 0.87 (0.50, 1.54) | 0.639 |
|  | Behaviour change | 1.18 (0.64, 2.18) | 0.600 | 1.19 (0.62, 2.27) | 0.601 |
| **Attitude** | Social norms | 2.15 (1.24, 3.72) | 0.006 ^a^ |  |  |
|  | Self-efficacy | 8.94 (5.00, 15.97) | <0.001 | 8.53 (4.77, 15.28) | <0.001 |
|  | Intention | 2.40 (1.43, 4.03) | 0.001 | 1.44 (0.81, 2.56) | 0.211 |
|  | Behaviour change | 1.47 (0.83, 2.61) | 0.190 | 1.07 (0.55, 2.06) | 0.852 |
| **Social norms** | Self-efficacy | 1.74 (1.11, 2.72) | 0.015 ^a^ |  |  |
|  | Intention | 1.63 (1.06, 2.49) | 0.025 ^a^ | 1.45 (0.93, 2.25) | 0.102 |
|  | Behaviour change | 1.19 (0.74, 1.91) | 0.466 | 1.03 (0.63, 1.69) | 0.911 |
| **Self-efficacy** | Intention | 3.12 (2.02, 4.84) | <0.001 |  |  |
|  | Behaviour change | 1.69 (1.05, 2.72) | 0.031 ^a^ | 1.28 (0.77, 2.13) | 0.339 |
| **Intention** | Behaviour change | 3.35 (2.08, 5.38) | <0.001 |  |  |

^a^ Becomes non-significant after Holm adjustment.
